# Fucosyltransferase 9 promotes neuronal differentiation and functional recovery after spinal cord injury by suppressing the activation of Notch signaling: Fucosyltransferase 9 promotes neuronal differentiation

**DOI:** 10.3724/abbs.2023138

**Published:** 2023-09-06

**Authors:** Jiewen Chen, Xiaolin Zeng, Wenwu Zhang, Gang Li, Haoming Zhong, Chengzhong Xu, Xiang Li, Tao Lin

**Affiliations:** 1 Department of Orthopedics and Traumatology Zhujiang Hospital Southern Medical University Guangzhou 510280 China; 2 Department of Spine Surgery The First Affiliated Hospital Sun Yat-sen University Guangzhou 510080 China

**Keywords:** neural stem cells, neuronal differentiation, neural stem cell transplantation, Notch, Wnt, fucosyltransferase

## Abstract

Individuals with spinal cord injury (SCI) suffer from permanent disabilities such as severe motor, sensory and autonomic dysfunction. Neural stem cell transplantation has proven to be a potential strategy to promote regeneration of the spinal cord, since NSCs can produce neurotrophic growth factors and differentiate into mature neurons to reconstruct the injured site. However, it is necessary to optimize the differentiation of NSCs before transplantation to achieve a better regenerative outcome. Inhibition of Notch signaling leads to a transition from NSCs to neurons, while the underlying mechanism remains inadequately understood. Our results demonstrate that overexpression of fucosyltransferase 9 (Fut9), which is upregulated by Wnt4, promotes neuronal differentiation by suppressing the activation of Notch signaling through disruption of furin-like enzyme activity during S1 cleavage. In an *in vivo* study, Fut9-modified NSCs efficiently differentiates into neurons to promote functional and histological recovery after SCI. Our research provides insight into the mechanisms of Notch signaling and a potential treatment strategy for SCI.

## Introduction

Individuals with spinal cord injury (SCI) suffer from permanent disabilities such as severe motor, sensory and autonomic dysfunction [1]. The World Health Organization estimates that between 250,000 and 500,000 cases are reported each year [2]. Surgical decompression, supportive therapies and rehabilitation protocols improve functional outcomes in patients [3]. Neurological dysfunction and failed function recovery are resulted from axonal and cellular damage, followed by regenerative failure [4]. There is still no efficient treatment to restore the integrity of the spinal cord itself [1]. Drugs and interventional procedures such as surgical treatment have been two pillars of most diseases for a long time, while biotherapies such as stem cell transplantation have emerged as the third most potent player over the past decades [ 5, 6]. Neural stem cell transplantation has proven to be a potential and feasible strategy to promote spinal cord regeneration after SCI in previous early-phase clinical trials [ 7, 8]. NSC transplantation provides new neurons to promote the formation of neuronal networks and functional connectivity [9]. However, stem cells transplanted into the spinal cord mainly differentiate into astrocytes, which are disadvantaged for tissue repair and result in poor functional recovery [10]. Therefore, it is necessary to optimize the neural-oriented differentiation of stem cells before transplantation.

Notch signaling is robust in most tissues and can regulate cell proliferation, acquisition of specific cell fates, and activation of differentiation programs [11]. The transmembrane protein Notch is modified and releases the Notch intracellular domain (NICD) from the transmembrane region to the nucleus [12]. Then, NICD combines with the DNA-binding protein RBPj and activates the Notch effectors Hes family bHLH transcription factor 1 (Hes1) and Hes5 [13]. In the mammalian nervous system, inhibition of Notch signaling leads to a transition from NSCs to neurons, while activation of Notch signaling significantly promotes the proliferation of neural stem cells [ 13, 14]. Thus, Notch signaling is important in neurodevelopment.

According to our previous publication, Wnt4 can promote neuronal differentiation by suppressing the Notch signaling pathway, but the molecular mechanism is still elusive [15]. Fucosyltransferase 9 (Fut9) belongs to the glycosyltransferase family, which catalyzes the last step in the biosynthesis of some oligosaccharides, such as Lewis X (LeX) antigen [ 16, 17]. Fut9 plays an important role in neurite formation and outgrowth [18]. The interaction between glycosyltransferase and Notch signaling has been described in a previous study [ 19, 20]. O-fucosyltransferase 1 (POFUT1), a member of the glycosyltransferase family, fucosylates the epidermal growth factor (EGF)-like domains of Notch and its ligands, which is a crucial step for the activation of Notch signaling [ 21, 22]. However, whether Fut9 can promote neuronal differentiation and the effect on the Notch signaling pathway during neurogenesis are still unclear. The aim of this study was to examine whether Fut9 can promote neural-oriented differentiation through the Notch signaling pathway.

## Materials and Methods

### Animals

Female Sprague-Dawley rats were obtained from the Experimental Animal Center of Sun Yat-Sen University (Guangzhou, China). All procedures were approved by the Animal Care and Use Committee of Sun Yat-sen University and were conducted in accordance with the Guide to the Care and Use of Experimental Animals by the National Research Council (1996, USA). The fetal brains of embryonic day 14 from pregnant SD rats were used for NSC cell isolation, and adult SD rats were used to establish an SCI animal model.

### NSC isolation and culture

NSCs were prepared from the fetal brains of embryonic day 14 rats, which were extracted from pregnant Sprague-Dawley rats. Briefly, rats were anaesthetized with 1% pentobarbital sodium (40 mg/kg) and sacrificed by CO _2_ asphyxiation. The brain tissue was dissected mechanically and dissociated in phosphate buffered saline (PBS). Then, the cell suspension was centrifuged at 200 *g* for 5 min. The supernatant was discarded, and the cell aggregation was resolved to a single-cell suspension. NSCs were plated into a T25 culture flask (Corning, Acton, USA) containing Dulbecco’s modified Eagle’s medium/F12, 2% B27, 1% penicillin/streptomycin, 1% L-glutamine (Thermo Fisher Scientific, New York, USA), 20 ng/mL FGF-2, and 20 ng/mL epidermal growth factor (Peprotech, Rocky Hill, USA). NSCs were cultured at 5% CO _2_ and 37°C and passaged via Accutase (Thermo Fisher Scientific) with weekly digestion. All NSCs used throughout this study were between passages 2 and 4.

Cells were dissociated and plated in 12-well plates coated with 0.01% poly-L-lysine (Phygene, Fuzhou, China) at a density of 5×10 ^4^ cells/well in neural-differentiated medium which consisted of Neurobasal medium (Gibco, Grand Island, USA), 2% B27, 1% penicillin/streptomycin, and 1% L-glutamine.

### Lentiviral vector construction and transduction

The lentiviral vectors carrying green fluorescent protein (GFP) with a sequence specifically overexpressing the *Fut9* gene were constructed by GeneChem (Shanghai, China). The oligonucleotides were ligated to hU6-MCS-CBh-gcGFP-IRES-puromycin (GeneChem) and had the following sequence: 5′-ACCTTACATCCTGCCAAGCC-3′. Control lentiviral vectors used a nontargeted sequence consisting of the same oligonucleotide bases, which were rearranged as indicated: 5′-TTCTCCGAACGTGTCACGT-3′. The vectors and corresponding packaging plasmids were cotransfected into 293T cells using Lipofectamine 2000 (Invitrogen, Carlsbad, USA). The medium was changed to complete medium after 8 h of incubation.

The supernatant was harvested from 293T cells after 48 h, filtered with a 0.45-μm pore size filter, and concentrated by ultracentrifugation at 96,500 *g* for 2 h at 4°C. After resuspension, 293T cells were transduced with serially diluted lentivirus. Then, the labelled 293T cells were counted to calculate the viral titer, and high-titer recombinant lentiviral vectors carrying *Fut9* were harvested 4 days later.

Cells in log phase were plated at a concentration of 1×10 ^5^ cells/well in six-well plates. Cells were transduced with the control lentivirus, Fut9-overexpressing lentivirus (LV-Fut9), in MEM-α (Gibco) with 10% FBS. Polybrene was added to improve the transduction efficiency at a concentration of 10 mg/mL. The medium was replaced by fresh complete medium after 8 h. Cells were harvested for injection of the SCI model and cell experiments after transduction.

### RNA-seq

Total RNA was extracted using a Trizol reagent kit (Invitrogen) according to the manufacturer’s protocol. Then, the enriched mRNA was fragmented into short fragments using fragmentation buffer and reverse transcribed into cDNA by using the NEBNext Ultra RNA Library Prep Kit for Illumina (NEB#7530; New England Biolabs, Ipswich, USA). The resulting cDNA library was sequenced using Illumina NovaSeq6000 by Gene Denovo Biotechnology (Guangzhou, China). To obtain high-quality clean reads, reads were further filtered by fastp [23] (version 0.18.0). DESeq2 [24] software was used to identify DEGs between two different groups (false discovery rate (FDR)<0.05 and absolute fold change ≥ 2).

### Real-time quantitative reverse transcription PCR (qRT-PCR)

Total RNA was obtained from NSCs using a Trizol reagent kit according to the manufacturer’s protocol, and 2 μg of total DNA-free RNA was used to synthesize cDNA using Evo M-MLV RT Premix for qPCR (Accurate Bio, Changsha, China). qPCR was performed on an ABI StepOnePluse PCR detection system (Applied Biosystems, Foster City, USA) using SYBR Green PCR Master Mix (Takara, Dalian, China). The process was as follows: 95°C for 5 min, followed by 45 cycles (95°C for 10 s and 60°C for 30 s). Parallel amplification of *GAPDH* was used to normalize gene expression. qPCR primer sequences are listed in Table 1 .

**Table:** **Table 1** Sequences of primers used in this study

Gene	Forward primer (5′→3′)	Reverse primer (5′→3′)
*Fut9*	CCGCCGATCTTTCCTACTCC	CACATTCTAAATCGATCCCAGCA
*NF200*	ATTGCTGAACGCTCCACGTA	GGACTTCAGTGGACAGCACA
*β3-tubulin*	AGCTCACCCAGCAGATGTTC	AAGGTGGCTAAAACGGGGAG
*MAP2*	CCAACACTAGCGGAACGATG	TGGTTTTACGGGTTGGCTGT
*Hes1*	TGGAATAGCGCTACCGATCAC	CGGAGGTGCTTCACTGTCAT
*Hes5*	CATCAACAGCAGCATTGAGCA	CGAAGGCTTTGCTGTGCTTC
*Ngn1*	GGGAGGGATACCTGACCACT	GGGTCAGTTCTGAGCCAGTC
*Ngn2*	CAAAGGATTATGGCGTGCGG	CATGAAGCGATCCTCCCTCC
*NeuroD1*	AGCCCCCTAACTGATTGCAC	CCCGGGAATGGTGAAACTGA
*NeuroD2*	GTACCCCGCCTGGTAGAGAT	GCGTTTCGATCTTGGACAGC
*GAPDH*	TGTGAACGGATTTGGCCGTA	GATGGTGATGGGTTTCCCGT

### Western blot analysis

Cells were lysed in RIPA buffer and total protein was extracted. Then, the protein concentration was determined by BCA assay. A 10% SDS-PAGE gel (Asegene, Guangzhou, China) was loaded with 20 μg of total protein, and the separated proteins were transferred by electroblotting to PVDF membranes. The membranes were blocked with 5% nonfat dry milk in TBST (50 mM Tris, pH 7.6, 150 mM NaCl, and 0.1% Tween 20) and then incubated with the primary antibody overnight at 4°C. Antibodies included anti-Fut9 (ab176794; 1:500; Abcam, Cambridge, UK), anti-NF200 (18934-1-AP; 1:1000; Proteintech, Chicago, USA), anti-β3-tubulin (ab18207, 1:1000; Abcam), anti-MAP2 (AF4081; 1:1000; Affinity Biosciences, Cincinnati, USA), anti-Hes1 antibody (ab108937; 1:1000; Abcam), anti-Hes5 antibody (ab194111; 1:1000; Abcam), anti-β-tubulin (ab179511; 1:1000; Abcam), and anti-GAPDH (AF7021; 1:1000; Affinity Biosciences). Blots were washed three times with TBST for 5 min each time, and membranes were incubated with HRP-conjugated goat anti-rabbit IgG antibody (ab6721; 1:5000; Abcam) for 1 h at 25°C. Then, the protein bands were visualized with Immobilon Western Chemiluminescent HRP Substrate (Sigma-Aldrich, St Louis, USA).

### Immunofluorescence microscopy

Cells were fixed in 4% paraformaldehyde (PFA) for 30 min and permeabilized with 0.5% Triton X-100 for 30 min. After 3 times wash with PBS, cells were blocked with 5% normal goat serum for 1 h. The cells were incubated overnight at 4°C with primary antibodies against the following antigens: anti-NF200 (18934-1-AP; 1:200; Proteintech, Chicago, USA), anti-β3-tubulin (1:1000), and anti-MAP2 (1:200). After three times wash with PBS, the primary antibodies were probed with Alexa Fluor 594 goat anti-rabbit (ab150080; 1:500; Abcam) and Alexa Fluor 488-conjugated goat anti-mouse (ab150117; 1:500; Abcam) secondary antibodies for 1 h at 25°C. Finally, the coverslips were washed with PBS three times and mounted using Prolong Gold Antifade Reagent containing 4′,6-diamidino-2-phenylindole (DAPI) (ab104139; 1:1000; Abcam). Cells were observed with an Axio Observer Z1 fluorescence microscope (Zeiss, Oberkochen, Germany).

### Surgical procedure and cell transplantation

Spinal cord surgery was performed on female Sprague-Dawley rats (180–220 g). Rats were divided into four groups ( *n*=5): the sham group, the SCI group, the LV-Con group, and the LV-Fut9 group. Cells transduced with the control lentiviruses or Fut9-overexpressing lentivirus (LV-Fut9) were collected for transplantation. Briefly, animals were anaesthetized with 1% pentobarbital sodium (40 mg/kg). The muscle and fat were cleared from thoracic vertebrae 9 and 10 (T9, T10), and the spinal cord was exposed via laminectomy. In the sham group, rats were operated on with laminectomy only; in the other three groups, rats were subject to surgery with complete transection of the T9‒T10 spinal cord, and 2 mm of spinal cord was removed. Then, 5 μL of NSCs at a density of 1×10 ^5^ cells/μL were implanted immediately rostral and caudal to the injured site using a microsyringe. Then, the muscle and skin of all rats were sutured, and penicillin was intraperitoneally injected daily for 1 week to protect against infection and dehydration. Postsurgical care of SCI rats included manual bladder expression twice daily until functional restoration.

### Furin-like enzyme activity assay

Fluorogenic furin substrate peptide (Pyr-Arg-Thr-Lys-Arg-AMC; Univ, Shanghai, China) was used to measure furin-like proprotein convertase activity. The experiment was performed as described in previous studies [ 25, 26]. Briefly, Cell lysates were thawed on ice and diluted 2-fold using 5× lysis/reaction buffer, then 20 μL was added to black opaque 96-well plates (Corning, Corning, USA) containing 70 μL of ultrapure water. The plates were then incubated 15 min at 37°C in an Infinite M1000 microplate reader (TECAN, Männedorf, Switzerland) prior to addition of 10 μL of 1 mΜ furin fluorogenic substrate peptide, which was pre-warmed for 30 min at 37°C whilst protected from light. Fluorescence intensity was measured immediately (with excitation at 355 nm and emission at 460 nm, over 1.0 s) using a DMI8 fluorescence microscope (Leica, Heidelberg, Germany) and repeated typically every 3 min for up to 4 h.

### Basso, Beattie and Bresnahan (BBB) testing

Postinjury motor behaviour was assessed via the BBB locomotion scale. Briefly, the scale (0‒21) represents sequential recovery stages and categorized combinations of rat joint movement, hindlimb movements, stepping, forelimb and hindlimb coordination, trunk position and stability, paw placement and tail position. A score of 21 indicates complete motor function, while 0‒1 indicates complete spinal cord transection. The higher score represents the better function.

### Histological analysis

At 8 weeks after SCI, all rats were deeply anaesthetized with an adequate dose of 1% pentobarbital sodium (40 mg/kg) and were transcardiac perfused with 250 mL of 0.9% normal saline. Animals were perfused with 4% paraformaldehyde (PFA) in 0.1 M phosphate-buffered saline (PBS, pH 7.4). The T8‒T12 cord segments were dissected based on the dorsal spinal root count to evaluate tissue repair roundly, postfixed overnight in 4% PFA, and soaked at 4°C overnight in 10% sucrose followed by 30% sucrose. The specimens were embedded in optimal cutting temperature compound, frozen at ‒20°C and sliced at a thickness of 20 μm in the longitudinal or transverse plane. To visualize the inflammatory cavity area around the lesion, animals ( *n*=5 per group) were sacrificed for hematoxylin-eosin (HE) staining. The T8‒T12 longitudinal spinal cord sections from each group were stained with HE according to standard protocols and observed under a bright field microscope [27]. To count the surviving neurons, animals ( *n*=5 per group) were sacrificed, and transverse sections of the injured spinal cord were used to stain neurons with Nissl [28]. Briefly, sections at 2 mm rostral and caudal to the lesion epicentre were counted for each rat. The numbers of positively stained cells were counted and averaged per section in a blinded manner.

### Statistical analysis

Data are expressed as the mean±standard deviation (SD). No test to identify outliers was performed. No sample size calculations were carried out in this study. All statistical analyses were performed with SPSS 25.0 software (SPSS, Chicago, USA) using one-way ANOVA with Bonferroni’s *post hoc* test. *P*<0.05 was considered statistically significant.

## Results

### Wnt4 upregulates Fut9 expression through the β-catenin signaling pathway

**Figure 1:**
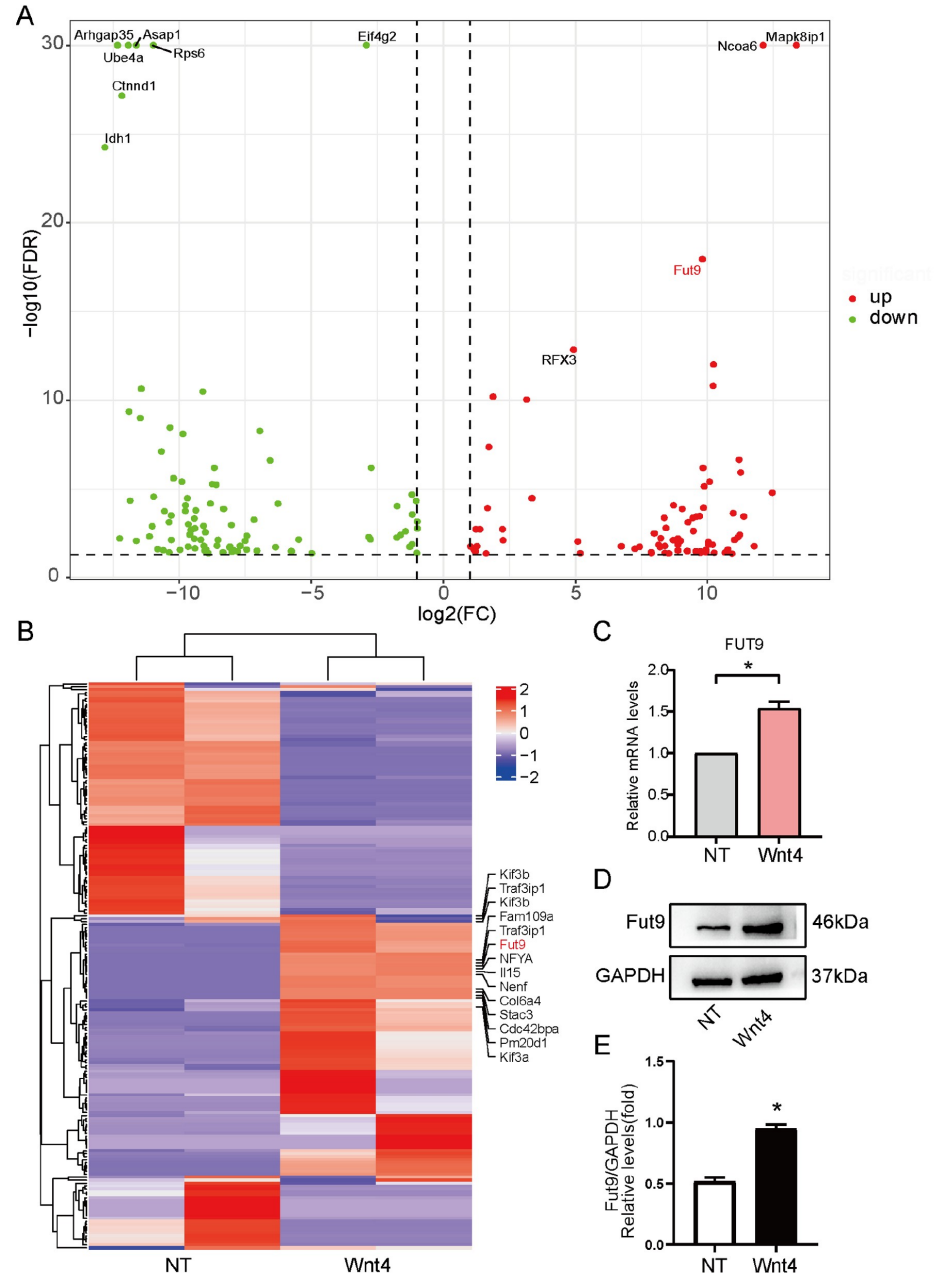
Figure 1 Fut9 is upregulated in the process by which Wnt4 promotes neuronal differentiation in NSCs (A,B) RNA sequencing of NSCs treated with Wnt4 for 3 days. (A) Volcano plots of differentially expressed genes (DEGs) in NSCs treated with Wnt4 in comparison with the untreated group. (B) Heatmap of DEGs of NSCs treated with Wnt4 in comparison with the untreated group. (C,D) RT-qPCR and western blot analysis of Fut9 expression in NSCs stimulated with Wnt4 for 3 days. (E) Quantification of Fut9 protein expression by western blot analysis. Data are presented as the mean±SD from one representative experiment of three independent experiments performed in triplicate. *P<0.05 compared with the untreated group.

Previous studies have demonstrated that the Wnt4 protein promotes neuronal differentiation and that Wnt4-modified neural stem cells promote therapeutic benefits *in vivo* [ 15, 29]. To further explore the gene regulatory mechanism triggered by Wnt4, mRNA-seq was performed. The heatmap shows the differentially expressed genes (DEGs). With the addition of Wnt4 (10 ng/mL for 7 days), 168 DEGs were observed, with 76 upregulated and 92 downregulated genes ( Figure 1A). Among these genes, Fut9 was significantly upregulated in both the heatmap and volcano map in the Wnt4-treated group ( Figure 1B). Similar to our mRNA-seq results, the expression of Fut9 was increased in the Wnt4-treated group compared with that in the untreated group at both the mRNA and protein levels ( Figure 1C‒E). These results indicated that Fut9 may be involved in neuronal differentiation by Wnt4.

**Figure 2:**
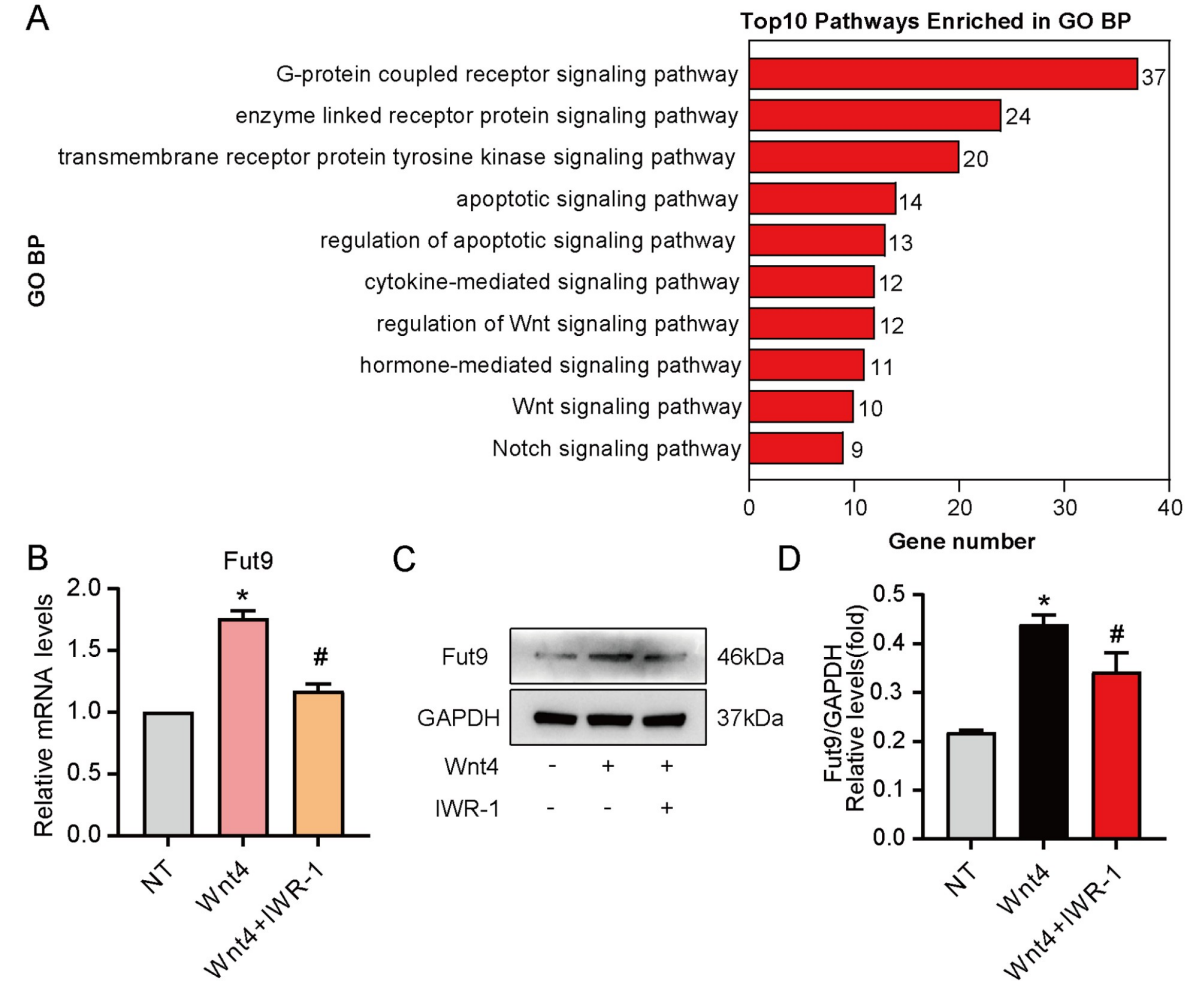
Figure 2 Wnt4 upregulates Fut9 expression through the β-catenin signaling pathway (A) GO pathway enrichment analysis of the differentially expressed genes between the NT group and Wnt4 group. (B,C) RT-qPCR and western blot analysis of Fut9 expression in NSCs treated with a specific pathway inhibitor and then stimulated with Wnt4 for 3 days. (D) Quantification of Fut9 protein expression by western blot analysis. Data are presented as the mean±SD from one representative experiment of three independent experiments performed in triplicate. *P<0.05 compared with the untreated group; #P<0.05 compared with the Wnt4 group. IWR-1: Wnt/β-catenin inhibitor.

To further determine the downstream signaling pathway by which Wnt4 regulates Fut9, GO pathway enrichment analysis of the differentially expressed genes between the NT group and Wnt4 group was performed. The results suggested that the Wnt signaling pathway might play an important role in the regulation of Fut9 ( Figure 2A). Therefore, we used a pharmaceutical inhibitor (IWR-1; 10 μM for 24 h) to inhibit the activation of canonical Wnt/β-catenin before treatment with Wnt4. The qPCR and western blot analysis results showed that the expression of Fut9 was significantly decreased by inhibiting the activation of β-catenin signaling ( Figure 2B‒D).

These results suggested that Wnt4 upregulates Fut9 expression through the β-catenin signaling pathway.

### Overexpression of Fut9 promotes NSC neuronal differentiation

**Figure 3:**
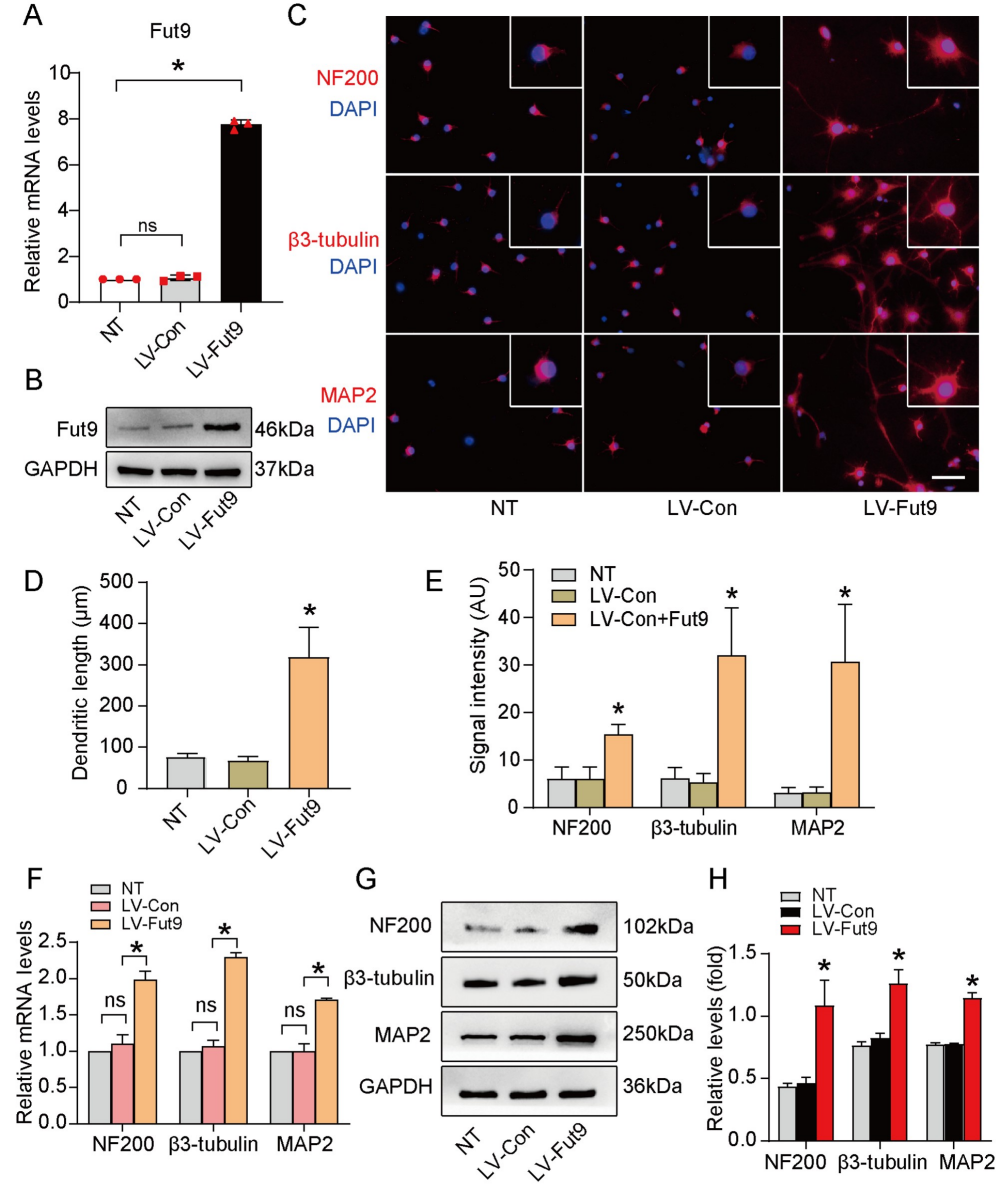
Figure 3 Overexpression of Fut9 promotes neuronal differentiation (A,B) mRNA and protein expression levels of Fut9 in the NT, LV-Con, and LV-Fut9 groups. (C) Immunofluorescence staining for neural-differentiated markers of NSCs in the NT, LV-Con, and LV-Fut9 groups. Scale bar: 100 μm. (D,E) Quantification of dendritic length and neural-differentiated marker-positive cells of NSCs. (F,G) mRNA and protein expression levels of NF200, β3-tubulin and MAP2 in the NT, LV-Con, and LV-Fut9 groups. (H) Quantification of neural-differentiated marker expression by western blot analysis. Data are presented as the mean±SD from one representative experiment of three independent experiments performed in triplicate. *P<0.05 compared with the nontreatment group and LV-Con group.

We further determined whether Fut9 can rescue the negative effect of the Notch signaling pathway on neuronal differentiation. LV-Fut9-transfected NSCs were collected for our *in vitro* experiment. The qPCR and western blot analysis results confirmed that Fut9 expression was successfully increased in NSCs ( Figure 3A,B). The immunofluorescence results showed that the intensity of NF200, β3-tubulin and MAP2 immunoreactivity and the dendritic length were significantly increased in the LV-Fut9 group compared with those in the NT group ( Figure 3C‒E). Similar effects on the expression of neuronal markers were observed at both the mRNA and protein levels ( Figure 3F‒H). These results indicated that overexpression of Fut9 could promote neuronal differentiation *in vitro* .

### Fut9 rescues the negative effect of Notch signaling to promote neuronal differentiation

**Figure 4:**
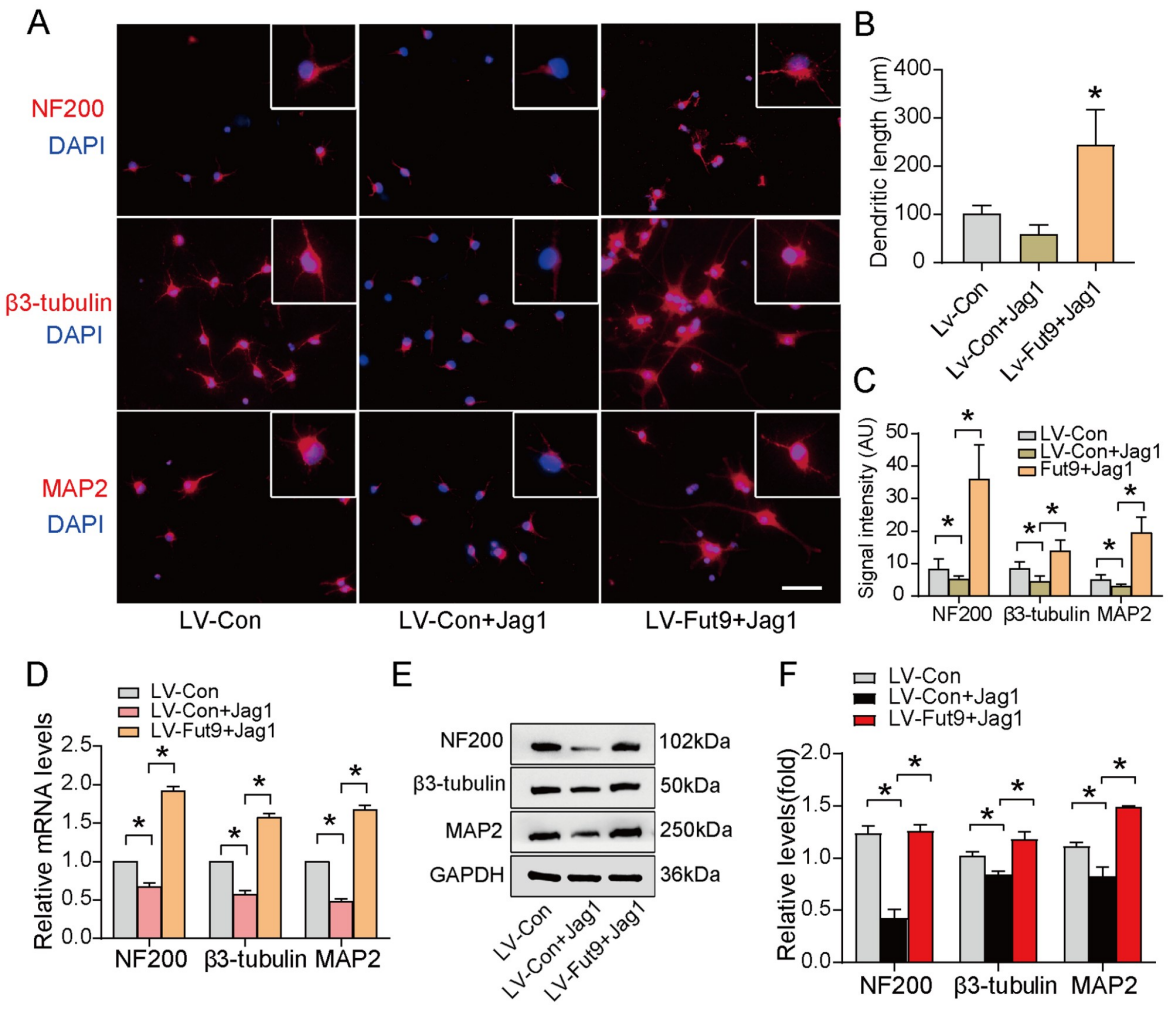
Figure 4 Fut9 rescues the negative effect of Notch signaling to promote neuronal differentiation (A) Immunofluorescence staining for neural-differentiated markers of NSCs in the NT, LV-Con+Jag1, and LV-Fut9+Jag1 groups. Scale bar: 100 μm. (B, C) Quantification of dendritic length and neural-differentiated marker-positive cells of NSCs. (D,E) mRNA and protein expression levels of NF200, β3-tubulin and MAP2 in the NT, LV-Con+Jag1, and LV-Fut9+Jag1 groups. (F) Quantification of neural-differentiated marker expression by western blot analysis. Data are presented as the mean±SD from one representative experiment of three independent experiments performed in triplicate. *P<0.05 compared with the LV-Con+Jag1 group.

The GO BP analysis showed that the Notch signaling pathway might contribute to neuronal differentiation regulated by Fut9 ( Figure 2A). Previous studies have demonstrated that activation of Notch signaling significantly promotes the proliferation of neural stem cells and suppresses neural differentiation [ 13, 14]. O-fucosyltransferase 1 (POFUT1) can activate Notch signaling by fucosylating the epidermal growth factor (EGF)-like domains of Notch and its ligands [21]. We further determined whether Fut9, which was upregulated by Wnt4, could rescue the negative effect of Notch signaling on the neuronal differentiation of NSCs. The immunofluorescence results showed that the intensity of NF200, β3-tubulin and MAP2 immunoreactivity was decreased by treatment with Jag1 (10 μM for 3 days), a Notch signaling agonist, suggesting that activation of Notch signaling suppressed neuronal differentiation and that this negative effect was abolished by overexpression of the *Fut9* gene ( Figure 4A‒C). Similar effects were observed at both the mRNA and protein levels ( Figure 4D‒F). These results demonstrated that Fut9 can rescue the inhibitory effect of Notch signaling to promote neuronal differentiation.

### Fut9 inhibits the furin enzyme activity of S1 cleavage to suppress the activation of the Notch signaling pathway

**Figure 5:**
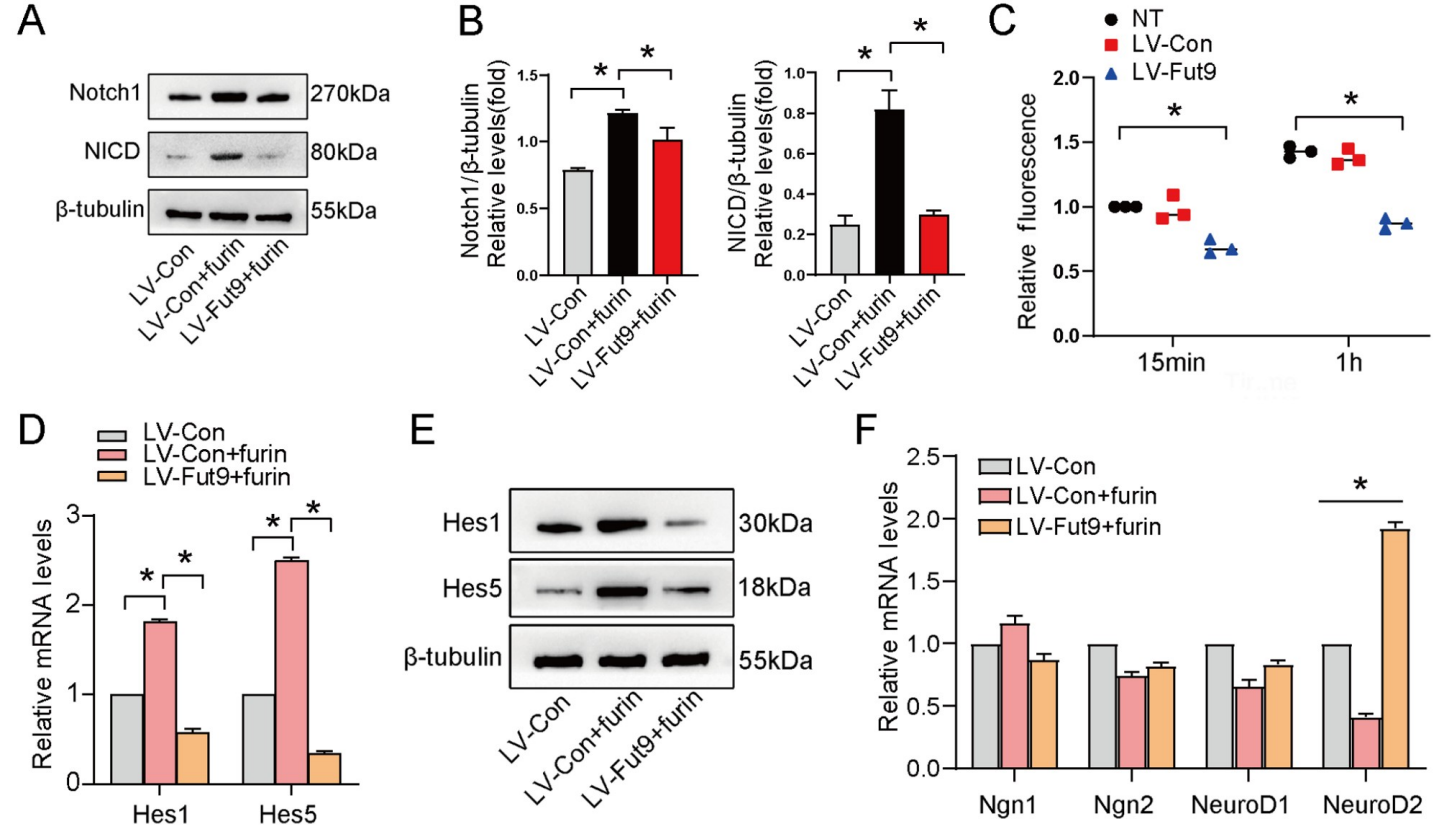
Figure 5 Fut9 inhibits the furin enzyme activity of S1 cleavage to suppress the activation of the Notch signaling pathway (A) Western blot analysis of Notch1 and NICD expression in NSCs treated with furin. (B) Quantification of Notch1 and NICD expression by western blot analysis. (C) Furin-like enzyme activity was measured in NSCs in different groups. Data are presented as the mean±SD from one representative experiment of three independent experiments performed in triplicate. *P<0.05 compared with the untreated group. (D,E) RT-qPCR and western blot analysis of Hes1 and Hes5 in the LV-Con, LV-Con+furin and LV-Fut9+furin groups. (F) RT-qPCR analysis of neural transcription factor expression in the LV-Con, LV-Con+furin and LV-Fut9+furin groups. Data are presented as the mean±SD from one representative experiment of three independent experiments performed in triplicate. *P<0.05 compared with the LV-Con+furin group.

The molecular mechanism by which Fut9 suppresses the Notch signaling pathway is unclear. To determine whether Fut9 suppresses the S1 cleavage of Notch, NSCs were treated with recombinant furin (20 U/mL) for 24 h. Western blot analysis results showed that NICD was significantly increased with furin treatment, while overexpression of Fut9 led to a decrease in NICD ( Figure 5A,B). We speculated that Fut9 may act on the furin-like convertase at the S1 site of Notch. Therefore, furin-like enzyme activity was detected in NSCs, and the results showed that the furin enzyme activity was decreased in the LV-Fut9 group compared with that in the untreated group ( Figure 5C). We further examined whether overexpression of Fut9 suppressed the expression of Hes1 and Hes5. As expected, Fut9 suppressed Hes1 and Hes5 gene expression at both the mRNA and protein levels ( Figure 5D,E). Moreover, we determined the neural transcription factors regulated by Fut9 and downstream signaling pathways. The qPCR results showed that NeuroD2 was significantly increased by Fut9 ( Figure 5 F). These results suggested that Fut9 suppressed the activation of the Notch signaling pathway by inhibiting the furin enzyme activity of S1 cleavage.

### Fut9-NSCs promote spinal cord repair *in vivo*

**Figure 6:**
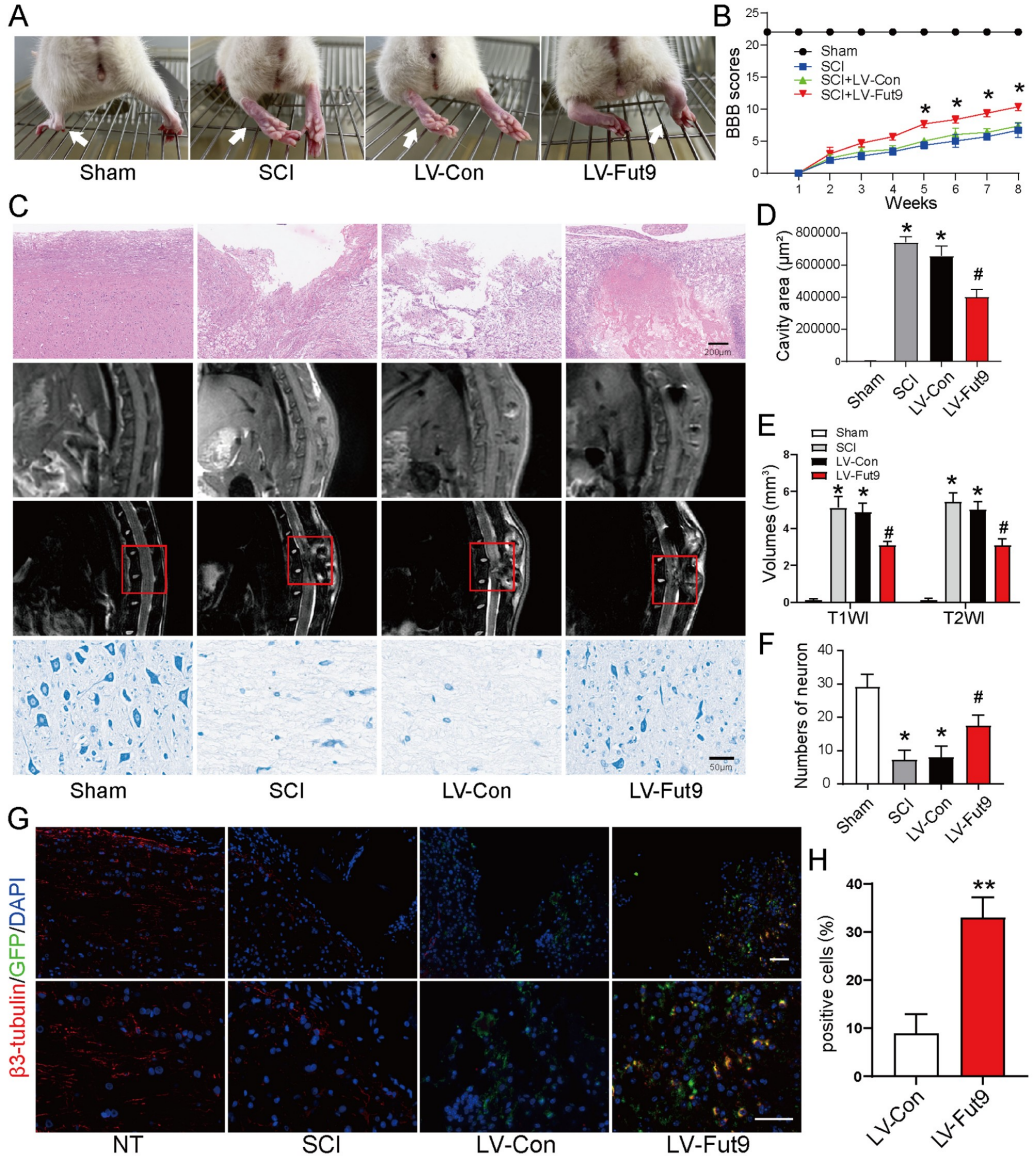
Figure 6 Fut9 promotes functional recovery and tissue repair (A) Images showing hind limb climb from Sham, SCI, LV-Con and LV-Fut9 groups at the eighth week postinjury; white arrows point to the hind limb. (B) BBB scores of the different groups. (C) H&E, MRI and Nissl staining analyses of the spinal cord in different groups. Sections at 2 mm rostral and caudal to the lesion epicenter were counted for each rat. (D,F) Quantification of H&E, MRI and Nissl staining analyses in different groups. Data are presented as the mean±SD. *P<0.05 compared with the sham group; #P<0.05 compared with the SCI group. (G) Immunofluorescence staining of the spinal cord in different groups. Scale bar: 50 μm. (H) Quantification of immunofluorescence staining. Data are presented as the mean±SD from two independent experiments. **P<0.001.

To investigate whether overexpression of Fut9 can enhance the therapeutic effect of NSC transplantation *in vivo*, LV-Fut9-transfected NSCs were injected into the spinal cord to assess the effect of LV-Fut9-transfected NSC transplantation on functional recovery. As shown in Figure 6A, the rats in the sham group grabbed and stepped easily using their hind limbs, whereas the rats in the SCI group could only keep their hind limbs dragged behind. As expected, the rats in the LV-Fut9 group could grab mildly and step slowly ( Figure 6A). Moreover, the BBB score at the eighth week after the operation showed that the hind limb locomotor activity in the LV-Fut9 group was markedly elevated compared with that in the SCI group ( Figure 6B).

We further investigated the spinal cord repair effect of Fut9-NSC transplantation *in vivo*. The lesion cavity in HE staining was used to detect spinal cord repair at 8 weeks after surgery, and the results showed that the total lesion cavity in the LV-Fut9 group was significantly smaller than that in the SCI group ( Figure 6C,D). Nissl staining was used to determine the ventral horn motor neurons at the lesion epicenter. The results showed that the number of surviving neurons in the LV-Fut9 group was significantly increased compared with that in the SCI groups ( Figure 6C,E). We used MRI to measure the volume of the injured sites. The results showed that the spinal cord was clearly separated by a gap after surgery, whereas the volume of the lesion was significantly decreased in the LV-Fut9 group ( Figure 6C,F). We further determined the differentiation status of the transplanted NSCs near the injury site. Immunofluorescence microscopy results showed that the number of GFP ^+^ β3-tubulin ^+^ cells was significantly increased in the LV-Fut9 group compared to that in the LV-Con group ( Figure 6G,H). These results suggested that Fut9 promotes the functional recovery and tissue repair of the spinal cord *in vivo*.

## Discussion

Traumatic spinal cord injury is a difficult problem that has devastating physical and social consequences for patients [ 1, 30]. There are no approved therapies to regenerate neural mobility and sensation [31]. Therefore, it is vital to find an agent that can improve the neuronal differentiation efficacy of neural stem cell transplantation. Since a previous study showed that fucosyltransferase promotes embryonic stem cell differentiation [32], we speculated that Fut9 might be a potential therapy for SCI.

In this study, we provided evidence for the function of Fut9 in neuronal differentiation after SCI. First, we proved that Wnt4 enhances the expression of Fut9 in NSCs through the canonical pathway ( Figures 1 and 2). Second, we confirmed that Fut9 inhibits Notch signaling in NSCs by suppressing the S1 cleavage of Notch1 (Figures 3‒ 5). Third, we demonstrated that Fut9-modified NSCs improves the therapeutic effect in spinal cord injury ( Figure 6).

The mRNA-seq analysis revealed that the expression of Fut9 was increased in the Wnt4-treated group, suggesting that Fut9 might play an important role in neuronal differentiation. We first confirmed that Wnt4 increases Fut9 expression through the β-catenin pathway ( Figures 1 and 2). Previous studies have shown that Fut9 is required for neurodevelopment [ 17, 33]. Gouveia *et al*. [18] reported that Fut9 is important for neurite outgrowth. Li *et al* [32] showed that Fut9 promotes embryonic stem cell differentiation through the cell recognition molecule L1. However, it is not clear whether Fut9 can promote neuronal differentiation of NSCs and improve the therapeutic effect of NSC transplantation. In this study, we first confirmed that Fut9 induces neuronal differentiation *in vitro* ( Figure 3). The underlying mechanism of Fut9-induced neuronal differentiation remains inexplicit. Previous studies have shown that the activation of Notch signaling significantly promotes the proliferation of neural stem cells, while inhibition of Notch signaling leads to a transition from NSCs to neurons [ 13 , 34, 35]. Activation of Notch signaling requires three cleavage and processing events of the Notch receptor, which is important for maintaining NSCs in a proliferative state [36]. S1 cleavage of the Notch receptor is necessary for Notch receptor maturation. Furin-like convertase is the protease for the S1 cleavage site [26]. S1 furin-like cleavage in the Golgi is regulated by Botch, and NICD is increased after treatment with furin [ 37, 38]. Moreover, there is an important interaction between fucosyltransferase and Notch signaling [39], and O-fucosyltransferase 1 (POFUT1) is required for Notch signaling [ 11, 40]. Our previous study showed that Wnt4 suppresses Notch signaling by downregulating NICD expression and inhibiting the binding between NICD and RBPj, ultimately promoting neuronal differentiation. However, the underlying mechanism was unclarified [15]. Therefore, we investigated whether Fut9, one of the DEGs of the Wnt4-treated group, is indispensable for the inactivation of Notch signaling. Our results revealed that Fut9 might promote neuronal differentiation by suppressing the activation of the Notch signaling pathway ( Figures 2A and 4). We found that Fut9 suppressed furin-like enzyme activity during S1 cleavage of Notch ( Figure 5) and finally activated Hes 1/5 transcriptional activities ( Figures 4 and 5). These results suggested that Wnt4 upregulates Fut9 expression, leading to a decrease in the furin-like enzyme activity of S1 cleavage and finally suppressing the activation of the Notch signaling pathway. Taken together, our findings revealed that Fut9 promotes neuronal differentiation by rescuing the inhibitory effect of Notch signaling on neuronal differentiation.

NSC transplantation has been one of the most promising preclinical therapeutic strategies in SCI over the last decade because NSCs can regenerate neural recruitment in the lesion [41]. It is necessary to optimize the differentiation of NSCs before transplantation to achieve a better regenerative outcome [42]. To further determine the role of Fut9 in neuronal differentiation and regeneration, we performed an *in vivo* experiment. Our results showed that locomotor recovery of hind limbs was improved after stem cell transplantation. Fut9-modified NSC transplantation partially repaired the injury site of the spinal cord ( Figure 6). In conclusion, our findings suggest that Fut9 promotes neuronal differentiation by suppressing the activation of Notch signaling in NSCs. Fu9-modified NSCs are a potential optimized method for stem cell transplantation.
